# Evaluation and students’ perception of a health equity education program in physical therapy: a mixed methods pilot study

**DOI:** 10.1186/s12909-024-05471-6

**Published:** 2024-05-01

**Authors:** Alexis A. Wright, Dominique Reynolds, Megan Donaldson

**Affiliations:** 1 Doctor of Physical Therapy Program, Department of Rehabilitation Sciences, Tufts University, Boston, MA 02111 USA; 2 Department of Rehabilitation Sciences, Medical University of South Carolina, Charleston, SC 29425 USA

**Keywords:** Doctor of physical therapy, Curriculum, Diversity, Health equity, Inclusion, Health professions

## Abstract

**Background:**

Health equity is a common theme discussed in health professions education, yet only some researchers have addressed it in entry-level education.

**Purpose:**

The purpose of this study is to serve as an educational intervention pilot to 1) evaluate students’ perception of the effectiveness of the DPT program in providing a foundation for health equity education, with or without the benefit of a supplemental resource and 2) establishing priorities for the program related to educating students on health inequities in physical therapy clinical practice.

A mixed method design with a focus-group interview was utilized to explore students’ perceptions of the DPT program's commitment to advancing health equity.

**Methods:**

A three-staged sequential mixed methods study was conducted. Stage 1 began with quantitative data collection after completing the DEI Bundle utilizing the Tripod DEI survey. Stage 2 involved identifying themes from the Tripod Survey data and creating semi-structured interview questions. Stage 3 consisted of a focus group interview process.

**Results:**

A total of 78 students completed the Tripod DEI survey upon completing 70% of the curriculum. Thirty-five students, eight core faculty, 13 associated faculty, and four clinical instructors completed the APTA DEI Bundle Course Series. According to the Tripod DEI Survey results, program stakeholders found the program’s commitment to DEI and overall climate to be inclusive, fair, caring, safe, welcoming, and understanding of individuals from different backgrounds, including a sense of student belonging where students feel valued and respected. Three themes emerged from the qualitative focus group interviews, including the value of inclusivity, health equity curricular foundations, and DEI in entry-level DPT education.

**Conclusions:**

This study highlights the value of incorporating health equity and DEI topics into curricula while fostering an incluse program culture.

## Introduction

### Background

Racial and ethnic disparities in healthcare are a longstanding and well-documented crisis in the United States [1]. A strategic goal of the American Physical Therapy Association (APTA) is to increase diversity, equity, and inclusion within the profession to serve society's health better. At its core, physical therapy is rooted in optimizing overall health and decreasing preventable illness and injury. Additionally, physical therapists are trained to be adaptive and respond to patients' social and environmental influences that impact health outcomes. These foundational traits uniquely position healthcare providers with the skills to respond to health inequities. Education and training for health providers are rarely studied to determine the effectiveness or implementation of the educational training [1, 2]. Specifically, diversity, equity, and inclusion (DEI) education training provides a basis to confront systemic racism and improve health equity, and physical therapy programs are being called to action [2]. However, the measurement of learners’ awareness and perceived effectiveness of educational interventions has lagged [1].

The literature review on this topic includes a study by the Institute of Medicine (IOM), which has provided recommendations for addressing and eliminating racial/ethnic disparities in healthcare. These recommendations include increasing healthcare providers’ awareness of racial/ethnic disparities in healthcare and educating health providers on health disparities, cultural competence, and the impact of race/ethnicity on clinical decision-making [3] A developing entry-level Doctor of Physical Therapy program intentionally designed curricula aligned with the IOM recommendations. Curricular topics were informed by the Clinical Prevention and Population Health Curriculum Framework, a product of the Healthy People Curriculum Task Force established in 2002 by the Association for Prevention Teaching and Research (APTR) [4]. Knowledge-based activities were designed to further awareness and understanding of the social determinants of health, health prevention, cultural awareness, health inequities, healthcare accessibility, systems thinking, and implicit and explicit bias among entry-level DPT students. The theoretical framework of the DPT curriculum is based on a theoretical framework of constructivism, which refers to the belief that learners actively construct knowledge by linking new information to what they have previously learned and by incorporating new experiences into their knowledge base and that learners’ knowledge structures are continually constructed and reconstructed [5].

Additionally, co-curricular educational activities were promoted throughout the program.

The theoretical framework for co-curricular educational activities is based on relational learning. Specifically, this model has been used for health promotion and inclusion [6, 7]. The co-curriculum does what the standard academic curriculum generally does not: it is developmental, transformative, and future-focused. For example, as a program, sessions were provided for learners to attend speaker sessions on DEI topics, apply for leadership roles (including the Diversity, Equity, and Anti-Racism (DEAR) Council), and engage in service activities, all grounded in an expectation of professional behaviors that encourage intellectual discussions on complex topics in an environment free of criticism, discrimination, harassment or any other emotional or physical harm.

The purpose of this study is to serve as an educational intervention pilot to 1) evaluate students’ perceptions of the effectiveness of the DPT program in providing a foundation for health equity education, with or without the benefit of a supplemental resource, and 2) establish priorities for the program related to educating students on health inequities in physical therapy clinical practice.

## Materials and methods

### Participants and study design

Determining the research question(s) is vital in the mixed research process. Research questions are pivotal in the mixed research process, which is interactive, emergent, fluid, and evolving [8]. As Leech and Onwuegbuzie [8] defined, “mixed methods research questions combine or mix both the quantitative and qualitative research questions necessitating the resulting data be collected and analyzed.” Mixed research sampling designs can be classified according to (a) the time orientation of the components (e.g., whether the qualitative and quantitative phases occur concurrently or sequentially) and (b) the relationship of the qualitative and quantitative samples (e.g., identical vs. parallel vs. nested vs. multilevel).

*Design: *To address the objectives of this study, a partially mixed-method design with a sequential and nested relationship was selected. The nested structure implies that individuals chosen for one phase of the study (qualitative focus group interviews) constitute a subset of those selected in the preceding phase (participants in the quantitative surveys) [8, 9]. Nonetheless, qualitative and quantitative research methodologies hold equal significance in this study's design and analytical approach.

*Sampling Strategy:* Participant enrichment refers to the mixing of qualitative and quantitative techniques for the rationale of optimizing the sample. Beginning with Phase 1, a total of 153 participants, including students (81) from the Class of 2022 (as pre-professionals) and 2) program faculty (16), associated faculty (36), and clinical instructors (20) (as post-professionals) were offered the option to participate in this mixed methods study. An email describing the purpose of the study was sent to all participants.

**Fig. 1:**
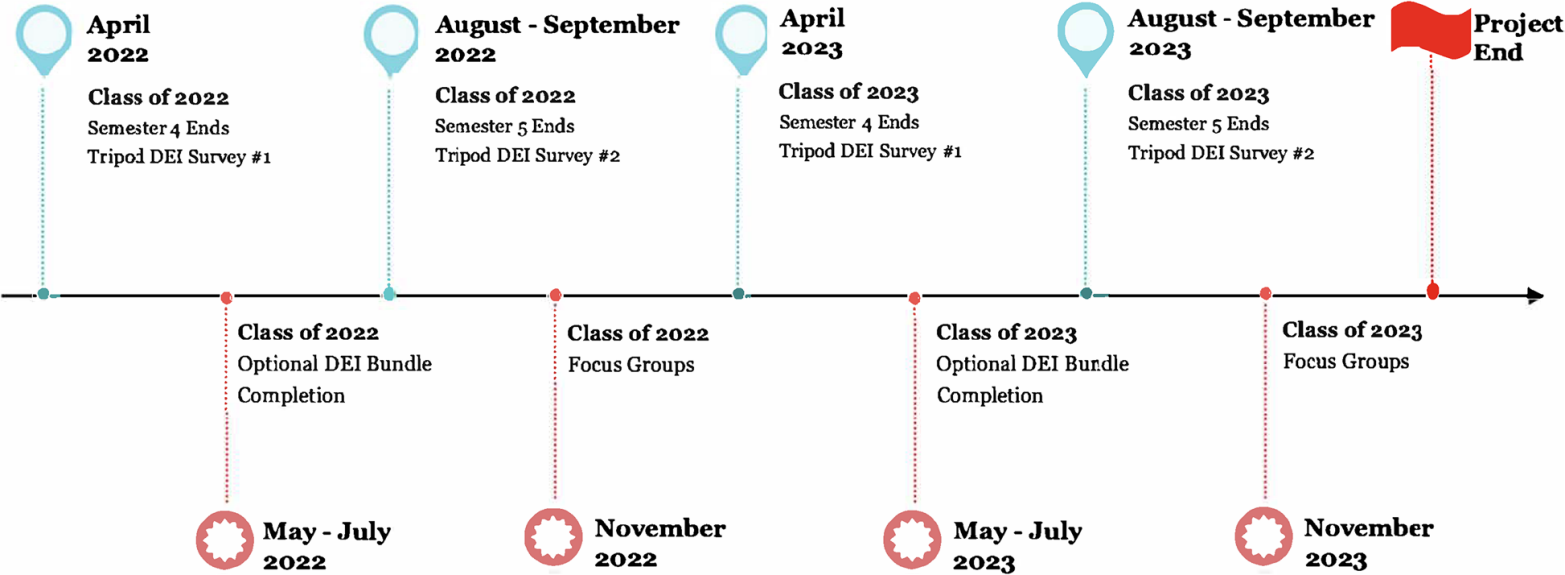
Timeline and Process for Study

Within mixed-method designs, instrument fidelity is essential and used by researchers to maximize the appropriateness and utility of the quantitative and qualitative instruments used in the study. These included the Tripod DEI survey, the APTA Diversity, Equity, and Inclusion (DEI) Bundle, and the qualitative semi-guided interview process. Stage 1 began with quantitative data collection after completing the Diversity, Equity, and Inclusion Bundle utilizing the Tripod DEI survey. Stage 2 involved identifying themes from the Tripod Survey data and creating semi-structured interview questions. Stage 3 consisted of the focus group interview process. See further details outlining the timeline and phases of the study in Fig. 1. Timeline and Process for Study.

The research implementation began with the quantitative survey, in which all students were surveyed using the Tripod DEI survey, which was deployed after semester 4 of the program, reflecting 70% completion of the curriculum [10]. Students were allowed to participate in the voluntary, supplementary APTA DEI Bundle beginning in Semester 5 [11]. Before participating in the APTA DEI Bundle, the Tripod DEI Survey was readministered to all students, program faculty, associated faculty, and clinical instructors who elected to participate [10]. Following completion of the APTA DEI Bundle, the Tripod DEI Survey was readministered a second time to all students, program faculty, associated faculty, and clinical instructors who completed the APTA DEI Bundle course series [10, 11]. The pre-test and post-test methodologies explored differences between adding the American Physical Therapy Association DEI Bundle to the program’s curriculum and co-curricular activities [11].

The study commenced once approval to conduct it was obtained from the Institutional Review Board at the university. After the submission was reviewed, the Tufts University IRB office determined that the proposed activity was not deemed human research as defined by DHHS and FDA regulations. (IRB ID:STUDY00002820).

### Research planning: quantitative study instrument

Tripod Education Partners works with programs to gather, organize, and report on student and teacher perspectives [10]. The Tripod DEI survey captures student perceptions of how diversity, equity, and inclusion issues play out in their school. The survey collects feedback from teachers about their experiences as teachers and perspectives about strengths and opportunities for improvement. Permission and funding for survey distribution were obtained before disseminating the survey.

The survey consisted of a total of 38 questions with eight distinct measures including 1) School commitment to DEI (*N* = 3), 2) School climate overall (*N* = 4), 3) School climate for DEI (*N* = 4), 4) Classroom teaching supporting DEI (*N* = 7), 5) Co-Curricular activities supporting DEI (*N* = 3), 6) Everyday discrimination by students (*N* = 6), 7) Everyday discrimination by teachers (*N* = 6), 8) Meaningful interactions across difference *N* = 5) (Tripod Education Partners,2019). School commitment to DEI is scored on a Likert scale from 1 (totally untrue) to 5 (totally true). School climate overall and DEI are scored as ordinal variables, with 2 being more favorable. Classroom teaching supporting DEI is scored on a Likert scale from 1 (none) to 5 (all). Co-curricular activities supporting DEI is scored on a Likert scale from 1 (my school doesn’t sponsor things like this) to 6 (very often). Everyday discrimination by students and teachers and meaningful interactions across differences are scored on a Likert scale from 1 (never) to 5 (very often).

The “overall sense of belonging” (*N* = 3) was scored on a Likert scale from 1 (totally untrue) to 5 (totally true).

The Tripod DEI survey development shows good construct validity and internal consistency [10]. Diverse student populations are at the center of the survey. Reports disaggregate findings by social identities across various groups, including but not limited to race, gender, and socioeconomic status. This breakdown allows programs to pinpoint groups of students reporting less-than-positive experiences and take action to address their needs.

### Research planning: description of the DEI training bundle

The optional training program was conducted through asynchronous electronic delivery of the APTA DEI bundle [11]. This program is a three-part series exploring foundational concepts related to diversity, equity, and inclusion and is led by Diana Lautenberger, MA, co-lead of the American Medical Colleges' leadership development seminar program. The three-part series utilizes a highly reflective approach whereby participants learn about identity, privilege, bias, and allyship as foundational pillars to achieving DEI. In addition, participants engage in self-reflection throughout the series to apply concepts to their clinical and personal lives to create more respectful and inclusive environments.

The series consists of three two-hour sessions: Part 1 – Unconscious Bias in the Health Professions; Part 2 – Power, Privilege, and Microaggressions; Part 3 – Responding to Microaggressions Through Allyship. The elements of this bundle listed objectives for the learners to 1) understand how their various identities carry social capital or power, 2) describe aspects of a dominant culture that advantage some and disadvantage others, and 3) utilize allyship and bystander intervention strategies that reduce harm to create more respectful and inclusive environments [11]. This program requires the completion of an assessment from the training. Viewers who completed all three sessions and scored at least 70% on each session's assessment (built into the modules) were also allowed to earn 0.6 CEUs (six contact hours) and a certificate of completion.

### Research planning: qualitative focus group interviews

Using an explanatory sequential mixed methods study, the qualitative portion aimed to further understand the students’ perceptions, establish priorities for the program related to educating students on health inequities in physical therapy clinical practice, and evaluate the effectiveness of adding the DEI Bundle. Based on the results of the quantitative portion of the study, two researchers created questions that would be used in the focus group interviews. The a priori semi-structured question guide in Table 1 was designed to allow emergent focus group discussion to explore concepts further.

**Table 1:** Semi-structured focus group interview guide

Semi-guided questions
Bias and Discrimination Questions (Quantitative topic): Qualitative Focus Group Questions · Do you believe that there is a bias within the program towards white students? Why or why not? · Do you believe that the program tries to learn about students/people from different backgrounds? Why or why not? · Do you believe this program is equitable to all families despite socioeconomic status? Why or why not?
Inclusivity and Meaningful Interactions (Quantitative topic): Qualitative Focus Group Questions · Tell me about your perception on the program’s communications with students with a different culture, religion, money, or sexual orientation other than your own? · Tell me about your thoughts and beliefs regarding classmate's communication with each other, including those with a different culture, religion, finances, or sexual orientation other than your own
Co-Curriculars and Classroom teaching (Quantitative topic): Qualitative Focus Group Questions · In what ways do you believe that the program embeds curriculum and learning activities focused on improving healthcare and improving access and difficulties identified by patients? · In what ways do you believe that the assignments and readings are representative of the population that you will treat as physical therapists? · What value do you find in learning about health equity in your entry-level educational program (curriculum) as it relates to systemic racism and physical therapy practice issues? · What is your perceived value of the Tufts DPT program resources to support DEI efforts through classroom teaching and co-curricular activities? · Do you feel you have developed the skills necessary to address health inequities in clinical practice? Why or why not
DEI Bundle (Quantitative topic): Qualitative Focus Group Questions · What is your perceived impact of the DEI Bundle in changing awareness, perception, and attitude-related health promotion programs to reduce health disparities? · What do you believe there is additional benefit to your learning on health equity from the DEI Bundle? · Did the addition of the DEI Bundle impact you or your feelings of preparation to develop the skills necessary to address health inequities in clinical practice? Why or why not? · How do you feel your participation in the DEI Bundle impacted your awareness, perception, and attitudes related to inequity in healthcare and healthcare education?

### Data analysis plan

#### Quantitative data collection and analysis

The data analysis program IBM SPSS 28.0 was utilized to store and analyze data from the Tripod DEI survey. For all the Tripod DEI survey subscales, items were summed, and scores were calculated. Descriptive statistics were utilized to calculate means, standard deviations, and 95% confidence intervals for each of the eight domains and Overall Sense of Belonging. Paired sample t-tests were conducted to compare pre-test and post-test scores. Summary independent samples t-tests compared the entire sample data (*N* = 81) to the post-DEI Bundle Series data.

#### Qualitative data collection and analysis

The semi-structured focus group interview guide questions (Table 1) were designed after the quantitative data collection was completed, and the data assessment revolved around concepts collected from the survey data.

A variety of data collection strategies were used, including (a) a mixture of open- and closed-ended items within the questionnaires that guided the focus group interview process, (b) a mixture of a priori (from the quantitative results) and additional emergent/flowing focus-group strategies through a semi-guided interview process. The Standards for Reporting Qualitative Research (SRQR) checklist was utilized for reporting.

Given the small sample size, no statistical software was utilized. Coding was used to assign labels to data segments to capture their meaning and allow comparison to identify themes or patterns. Both researchers used qualitative content analysis to systematically categorize transcribed content into topic areas from the thick descriptions provided. Qualitative fields were created to organize data by topic counts of language content areas (such as “DEI” and “belonging” quotes). The preliminary or open coding was done first and then refined to a higher level to reflect broader categories. All coding stages were done separately and then together to ensure improved accuracy. Then, the researchers used the comparison analysis and consensus approach to categorize and interpret data to identify patterns and content themes during the analysis. The analysis used a matrix table as a visual spreadsheet, where the rows represented participants, and the columns represented codes identified.

*Researcher characteristics and reflexivity:* The background and experience of the researchers could have influenced the research as two of the researchers had routine involvement with the participants within the study. The same researchers that conducted the study design and implementation conducted the focus group interviews via Zoom while participants were on clinical rotations. The focus-group interviews were audio-recorded and transcribed by an administrative coordinator who supported the faculty and had limited student interactions during daily work.

*Techniques to enhance trustworthiness:* The research team, consistent throughout the study, undertook the quantitative and qualitative data analysis. To maintain objectivity, they devised a set of a priori questions for interviews, steering clear of leading inquiries or interpretations. Subsequently, they conducted content analysis directly from transcriptions. Reflexivity strategies encompassed credibility checks via member validation and a post-session peer debriefing (between researchers), ensuring accuracy in focus group interviews. The research coordinator, unbiased to quantitative analysis, remained uninvolved in question formulation, solely providing session transcriptions for analysis. Furthermore, thick descriptions were provided, and qualitative counts of language content areas were evenly applied to promote the transferability of qualitative findings. By integrating these measures, the study aimed to mitigate inherent limitations in its design and bolster the credibility, transferability, dependability, and confirmability of its qualitative research, thus enhancing the trustworthiness and reliability of its findings.

## Results

### Quantitative analysis and results

A total of 78 students completed the Tripod DEI survey upon completing Semester 4 of the curriculum. A total of 42 students, eight core faculty, 16 associated faculty, and four clinical instructors elected to participate and complete the voluntary, supplementary pre-APTA DEI bundle Tripod DEI survey beginning Semester five. A total of 35 students, eight core faculty, 13 associated faculty, and four clinical instructors completed the APTA DEI Bundle Course Series. Thirty-two students, eight core faculty, 13 associated faculty, and four clinical instructors completed the post-APTA DEI Bundle Tripod DEI Survey.

#### Student results

Demographics of the full sample of 78 students can be found in Table 2.

**Table 2:** Demographic data of the full sample (*N* = 78)

Sex	Age (years)	Race/Ethnicity	Medically Underserved	Ever Homeless	First generation college student
Female (49) Male (29)	25.41 (21–54)	White (43) Black (2) Hispanic (5) Asian (14) Two or more (12) Not reported (2)	Yes (7) No (64) Not reported (7)	Yes (3) No (71) Not reported (4)	Yes (12) No (62) Not reported (4)

Survey results following the completion of Semester 4 are summarized below and reported as mean, standard deviation.

**School Commitment to DEI (1 = totally untrue; to 5 = totally true)**

Students generally found the program's commitment to DEI to be inclusive, fair, and understanding of individuals from different backgrounds (M = 4.1, SD = 0.9) or “mostly true”.

**School Climate Overall (1 = less favorable; 2 = favorable)**

Students reported the program's climate/culture as caring, respectful, safe, and welcoming (M = 2.0, SD = 0.1) where 2 is scored as caring, respectful, safe, and welcoming.

**School Climate for DEI (1 = less favorable; 2 = favorable)**

Students rated the program's climate/culture for DEI as “equally fair” to all students, regardless of their social identity (M = 1.9, SD = 0.2). This included questions related to race, ethnicity, sexual orientation, socioeconomic status, and gender where 2 is scored as equally fair to all students.

**Classroom teaching Supporting DEI (1 = none; 5 = all)**

Classroom teaching supporting diversity, equity, and inclusion rated “most but not all” (M = 4.1, SD = 0.8) faculty as having integrated material on different social identities, discussing issues of social inequality, and using student-centered teaching methods. This included questions related to helping students think about how to improve the world, leading discussions about why some people have difficult lives and other people have easier lives, connecting content from the classroom to problems or issues in the world as well as the student’s own life and interests, helping students think about how to improve other people’s lives, assigning readings or materials about people from different backgrounds or places, and taught about influential people from many different cultures.

**Co-Curricular Activities Supporting DEI (1 = my school doesn’t sponsor things like this; 6 = very often)**

With regards to co-curricular activities supporting diversity, equity, and inclusion, students reported on average that they “hardly ever” participated in a school-sponsored group for students of different racial, ethnic, socioeconomic, gender, sexual orientation, or ability groups; attended a school-sponsored event related to diversity, fairness, or inclusion; or participated in a program sponsored group working to make the world a better place (M = 3.3, SD = 1.0).

**Everyday Discrimination by Students (1 = never; 5 = very often)**

Students reported “never to hardly ever” regarding everyday discrimination by students regarding courtesy, respect, intelligence, being better than others, being bullied or threatened, and insults (M = 1.8, SD = 0.7).

**Everyday Discrimination by Teachers (1 = never; 5 = very often)**

Students reported “never to hardly ever” regarding everyday discrimination by faculty regarding courtesy, respect, intelligence, being better than others, being bullied or threatened, and insults (M = 1.4, SD = 0.6).

**Meaningful Interactions Across Differences (1 = never; 5 = very often)**

Students rated the program as “fairly often” with regards to meaningful interactions across differences, including honest discussions with other students whose religion was different from their own, whose families have more or less money than their own, whose culture is different from their own, and whose race is different from their own (M = 3.8, SD = 0.9).

**Belonging (1 = totally untrue; 5 = totally true)**

Finally, the students rated the program as “mostly true to totally true” concerning their sense of belonging in the program, whereby the student feels valued, respected, and a sense of belonging (M = 4.4, SD = 0.8).

### Comparison of tripod survey pre-post

Thirty-two students elected to participate and complete the APTA DEI Bundle Series with completed pre- and post-Bundle Series survey data. Demographic information on student participation in the DEI Bundle can be found in Table 3. After completing the APTA DEI Bundle Series, we found no significant difference in any of the eight domains or Sense of Belonging. We found no significant differences in any domain between the full sample (*N* = 78) and the post-DEI Bundle Series data sample (*N* = 32).

**Table 3:** Demographics of Students Participating in DEI Bundle Series (*N* = 32)

Gender	Age	Race/Ethnicity	Medically Underserved	Ever Homeless	First generation college student
Female (27) Male (5)	26.72 (21–54)	White (22) Black (1) Hispanic (1) Asian (6) Two or more (1) Not reported (2)	Yes (2) No (27) Not reported (3)	Yes (1) No (30) Not reported (1)	Yes (6) No (25) Not reported (1)

#### Post-professional stakeholder results

Twenty-five of our post-professional stakeholders elected to participate and complete the APTA DEI Bundle Series with completed pre- and post-Bundle Series survey data. After completing the APTA DEI Bundle Series, we found no significant difference in any of the eight domains or Sense of Belonging.

**School Commitment to DEI (1 = totally untrue; to 5 = totally true)**

Similarly, the post-professional stakeholders generally found the program's commitment to DEI to be inclusive, fair, and understanding of individuals from different backgrounds (M = 4.2, SD = 1.2).

**School Climate Overall (1 = less favorable; 2 = favorable)**

Post-professionals reported the program’s climate/culture overall as caring, respectful, safe, and welcoming (M = 2.0, SD = 0.0).

**School Climate for DEI (1 = less favorable; 2 = favorable)**

Post-professionals rated the program’s climate/culture for DEI as “equally fair” to all students, regardless of their social identity (M = 2.0, SD = 0.1). This included questions related to race, ethnicity, sexual orientation, socioeconomic status, and gender.

**Classroom Teaching Supporting DEI (1 = none; 5 = all)**

Post-professionals rated climate for DEI Classroom teaching supporting diversity, equity, and inclusion rated “most but not all faculty” (M = 3.8, SD = 1.0) as having integrated material on different social identities, discussing issues of social inequality, and using student-centered teaching methods. This included questions related to helping them think about how to improve the world, leading discussions about why some people have difficult lives and other people have easier lives, connecting content from the classroom to problems or issues in the world as well as the student’s own life and interests, helping students think about how to improve other people’s lives, assigning readings or materials about people from different backgrounds or places, and taught about influential people from many different cultures.

**Co-Curricular Activities Supporting DEI (1 = my school doesn’t sponsor things like this; 6 = very often)**

With regards to co-curricular activities supporting diversity, equity, and inclusion, post professionals reported on average that they “hardly ever participated” in a school-sponsored group for students of different racial, ethnic, socioeconomic, gender, sexual orientation, or ability groups; attended a school-sponsored event related to diversity, fairness, or inclusion; or participated in a program sponsored group working to make the world a better place (M = 2.9, SD = 1.0).

**Everyday Discrimination by Students (1 = never; 5 = very often)**

Post professionals reported “never to hardly ever” concerning everyday discrimination by students (M = 1.3, SD = 0.5).

**Everyday Discrimination by Teachers (1 = never; 5 = very often)**

Post professionals reported “never to hardly ever” concerning everyday discrimination by teachers (M = 1.4, SD = 0.5).

**Meaningful Interactions Across Differences (1 = never; 5 = very often)**

Post professionals rated the program as “fairly often” with regards to meaningful interactions across differences, including honest discussions with other students whose religion was different from their own, whose families have more or less money than their own, whose culture is different from their own, and whose race is different from their own (M = 3.1, SD = 0.9).

**Belonging (1 = totally untrue; 5 = totally true)**

Finally, the post professionals rated the program as “mostly true to totally true” regarding their sense of belonging in the program, whereby the student feels valued, respected, and a sense of belonging (M = 4.5, SD = 1.0).

### Result of qualitative focus group content analysis

From those participants completing the quantitative portion of the study, a nested sub-group of students (*n* = 9) volunteered to participate in the semi-structured focus group interview following the completion of the DEI Bundle. Demographic information on student participation in the interviews can be found in Table 4.

**Table 4:** Demographics of Students Participating in Semi-Structured Focus Group Interviews (*N* = 9)

Gender	Age	Race/Ethnicity	Medically Underserved	Ever Homeless	First generation college student
Female (8) Male (1)	25.33 (22–30)	White (6) Black (1) Asian (1) Two or more (1)	Yes (1) No (8)	No (9)	Yes (1) No (8)

There was a rich discussion with the interview guide around the topics 1) DEI with or without the training supplement related to health equity in physical therapy and 2) the program’s commitment to training students on topics associated with health equity. Three themes emerged from the qualitative focus group interviews based on the final qualitative content analysis.

#### Theme 1: student’s perceived value of inclusivity

Theme one was the value of inclusivity with three associated sub-themes of fairness, actions, and communication. In higher education, inclusivity is the ongoing process of improving the education system to meet the needs of all students, especially those in marginalized groups. Inclusivity involves reimagining educational services to cater to a diverse audience and making learning materials and teaching methods accessible to as many students as possible. This includes considering a range of diverse student identities, including race, gender, sexuality, and abilities. “*The program does make an effort, especially with adjuncts that we bring in, ableism talks, and people from different backgrounds speaking to us in classes on Zoom*.”

Additionally, providing sessions to improve inclusivity and communicating and demonstrating actions consistent with the value of inclusivity is essential to the participants. “*Being a member of the gay community, having a faculty in class that you feel you belong in and are not outcasted in is super important*.” Participants valued being included during activities and communicating support during school and personal life challenges. The participants recognized the challenge of finding people from different backgrounds who meet the expectations and specialties to teach within the program. They identified that, at times, visual diversity was limited within the core faculty but felt an intention of more inclusivity of race and ethnicity within the associated faculty roles or lecturers.

Within the value of inclusivity, there is also an inherent limitation to who can afford the DPT graduate-level program at a private university. Hybrid education offers more geographical convenience and reaches a more diverse student group; however, current students feel that money concerns could be a barrier to inclusivity, especially those in marginalized groups. *“Program doesn’t have control over the cost of tuition but does communicate what is available as far as opportunities for financial aid.”* However, they felt that communication about costs for the hybrid program and what financial aid was available was essential.

#### Theme 2: student’s perceived value of health equity curricular foundations

Theme two was the value of health equity curricular foundations with three sub-themes of representation in assignments, system resources, and practice issues. Health equity is the goal of helping people reach their highest level of health. It means everyone has a fair chance to achieve optimal health regardless of race, ethnicity, gender identity, or socioeconomic status. Health equity can be promoted through DEI initiatives, which focus on representing the acceptance and inclusiveness of people. The focus group reported health equity topics associated with race, social determinants, and access were satisfactorily addressed within the curriculum. However, there were opportunities to gain additional insights on improving formative activities to be more integrated with how health issues affect those with visual diversity. “*Activities within the program should also include skin tone other than white throughout systems-focused curriculum case studies, mannequins, and simulation/ standardized patients*.”

#### Theme 3: student’s perceive value of DEI in entry-level PT education

Lastly, one remaining theme specifically addressed DEI supplementation to the curriculum. Theme three is the value of DEI in entry-level physical therapy education, with three sub-themes emerging on the timing of content, planned redundancy of learning, and the limited value of a stand-alone DEI bundle. The students in the focus group had a consensus on their perceived confidence and appropriate knowledge of social determinants of health when working with the underserved population during their clinical education exposures. However, the focus group agreed with “*concerns about generalizing their feelings to all classmates, as some students may have had different experiences based on their final clinical education setting and exposure*.”

Additionally, according to the student perception, inclusivity and health equity values should be blended across the curriculum so that support and the training of those with different backgrounds can be promoted through DEI initiatives. Curriculum initiatives were given rich context regarding the program and curriculum that would be more “*inclusive and supportive of a health equity curricular track and activities threaded throughout the curriculum rather than a stand-alone module*.” There was a consensus from the focus group that mirrored the quantitative results that there was a perceived “*limited value in the DEI Bundle as a stand-alone module outside of the curriculum*.” Instead, the students preferred the curriculum designed to include the topics sufficiently within systems and population coursework.

#### Synthesis

The mixed methods analysis allows a better explanation of the student’s perceptions by blending the results from this study's qualitative and quantitative study portions. It was found in both portions of the study design that the program climate/culture is essential, especially as students relate inclusivity and accepting others when learning to value DEI from a health equity perspective. Students further strengthened their perceived value for their education and blended content topics across the curriculum as they related to health equity and diversity. Still, they found value when more than just content was presented. Students felt that there was a program culture, planned curriculum content, and co-curricular (outside of a class) support for health equity and inclusivity of the population's health care providers serve. As educators look to streamline variation in essential content across healthcare disciplines, utilizing a structured format (toolkit or bundle) could benefit students educationally but may be valued less by them.

## Discussion

Our study aimed to explore the students’ perceptions and establish priorities for the program regarding educating students on health inequities in physical therapy clinical practice.

Health equity is a common theme discussed in health professions education, yet only some have published the methods to address it in entry-level education. National organizations recommend that medical schools and health professions train students in the social determinants of health. This provides the opportunity to educate the next generation of healthcare professionals about sensitive yet essential issues.

Given the complexity of this topic, we utilized a three-staged sequential mixed methods approach to generate the results presented in this study. We found the program’s commitment to DEI and overall climate to be inclusive, fair, caring, safe, welcoming, and understanding of individuals from different backgrounds, including a sense of student belonging where students feel valued and respected. Additionally, the sample provided feedback on the educational approach and format, which was provided with the DEI Bundle. The modular-based curricular approach (not integrated through a course) was used in this study. Thus, the results of the APTA’s DEI Bundle should be considered, given the context of the study, regarding the curricular delivery and format as an “addition to” approach. Given this format, the DEI Bundle was insignificant due to the threaded curricular approach already within the program, as assessed on the Tripod DEI survey or qualitative focus group theme. This approach aligns with other recommendations for curriculum approaches to health equity [12] that integrate health equity content longitudinally and alongside other topics. The goal would be to eliminate views of health equity and healthcare as separate [13].

Limited studies explore health equity topics' style, content, and delivery through the healthcare professional’s entry-level educational program. However, the Association of American Medical Colleges recommends that medical educators expose their students to content about health disparities [14]. There are some challenges to implementing the recommendations [15], which are further complicated by the lack of recommendations regarding format, delivery, and the requisite degree of competency, which are poorly defined. Several resources are provided but not easily found across all health professions disciplines. However, several studies highlight the importance of health equity education, its impact on therapeutic relationships (trust and caring), and identify the consequences of implicit bias on patient adherence and outcomes [16].

Significant work must be done to unite all the health professions on strategies for implementing the health equity curriculum. However, an external resource strategy or modular-based approach could be effective, given limited resources and a lack of topic expertise within the program faculty. Still, it should be used with an integrated approach and placed intentionally within the curriculum design. It should have more opportunities for integration across courses, with case studies to facilitate thinking and reasoning and culminate in a competency type of assessment. Curriculum toolkits provided by professional associations may be one way to unite the disciplines to support health equity education in the health professions [17]. An excellent example of this approach is the American Academy of Family Practitioners Health Equity Curricular Toolkit, which has over 40 content experts [18, 19]. A threaded curriculum with a program culture and willingness to utilize health equity curriculum toolkits are essential for our next generation of health practitioners. These toolkits are resources for learning and reducing the variability in education [18]. Exploring outcomes associated with toolkits may be an option to begin to explore best practices in curriculum delivery to maximize learning outcomes and competency on health equity [20]. Lastly, any health equity resource or curricular approach should facilitate the exploration of some of the most pressing questions around social determinants of health, vulnerable populations, economics, and policy from an evidence-informed perspective.

### Limitations

There are several limitations that we would like to address. Within the quantitative portion of the study, the Tripod DEI survey adequately assessed overall student perception of the DPT program commitment to DEI; however, it may need more responsiveness surrounding the APTA DEI Bundle. Within any mixed methods design approach, it is important to address data fidelity during the qualitative portion. A non-investigator conducted both the survey distribution and outcome assessment; however, the focus group interviews were conducted by two study investigators. Additionally, both researchers are on the leadership team within the program, which may compromise the fidelity, trustworthiness, or sharing from the participants during this experience. It is a limitation in the study that the researchers also are involved in the education. Although a safe space and relational learning theory approach is utilized within the program, this may have limited some of the exploration of the topics/themes if the participants were sensitive. From what was shared in the focus groups, a non-investigator recorded and transcribed the data analysis portion. The second limitation of the qualitative focus groups was the limited number and need for more diversity within the sample. Specifically, the individuals who made time to participate in the qualitative focus group were not significantly diverse regarding their race or sex. The third limitation is the inability to identify the number of students who respond based on their participation in additional co-curricular activities to supplement their learning in DEI.

## Conclusions

However, significant work must be done to unite all the health professions on strategies for implementing health equity curricula. It was essential to gain insight from the students’ perception and establish priorities on the current curriculum and entry-level education program culture related to educating students on health inequities in physical therapy clinical practice. However, given limited resources and a lack of topic expertise for health equity content among program administrators and faculty, an external resource strategy or modular-based approach could be effective. However, based on our study, the program culture is important as it relates to DEI from a health equity perspective. It should be evident to students as we influence them to become the next generation of health professionals.

Lastly, the intentional curriculum design should have more opportunities for integration across courses with case studies and culminate in a competency type of assessment, even if an external resource is used. Resources are available to support health equity education in the health professions, including health equity curriculum toolkits, which provide free links and resources for learning and may help to reduce the variability in education [15]. Any health equity resource or curricular approach should facilitate faculty’s willingness to include some of the most pressing questions around social determinants of health, vulnerable populations, economics, and policy within their current or future developed curriculum. However, motivating incremental changes in entry-level professional teaching methods and working intentionally to integrate health equity into the clinic- and classroom-based environments are tangible next steps. Identifying best practices from education to implementation has yet to be well known, and this study only provided a pilot for future studies.

## Data Availability

The data supporting this study's findings are available from the corresponding author upon request.
